# Heating-up Synthesis of MoS_2_ Nanosheets and Their Electrical Bistability Performance

**DOI:** 10.1186/s11671-016-1385-x

**Published:** 2016-03-31

**Authors:** Xu Li, Aiwei Tang, Jiantao Li, Li Guan, Guoyi Dong, Feng Teng

**Affiliations:** Key Laboratory of Luminescence and Optical Information, Ministry of Education, School of Science, Beijing Jiao Tong University, Beijing, 100044 China; Hebei Key Laboratory of Optic-electronic Information and Materials, College of Physics Science and Technology, Hebei University, Baoding, 071002 People’s Republic of China

**Keywords:** MoS_2_, Nanosheets, Electrically bistable device, Charge transport

## Abstract

Molybdenum disulfide (MoS_2_) nanosheets were synthesized by using a simple heating-up approach, in which 1-dodecanethiol (DDT) was used not only as a sulfur source but also as the surface ligand. The sheet-like morphology was confirmed by the transmission electron microscopy (TEM) and atomic force microscopy (AFM) results, and the X-ray diffraction (XRD) patterns and Raman spectrum were employed to characterize the structure of the as-synthesized MoS_2_ nanosheets. The as-obtained MoS_2_ nanosheets blending with a polymer could be used to fabricate an electrically bistable device through a simple spin-coating method, and the device exhibited an obvious electrical bistability in the *I-V* curve. The charge transport of the device was discussed based on the organic electronic models.

## Background

In the past few decades, much attention has been paid to the two-dimensional (2D) nanomaterials due to their surprising unique physical, electrical, and chemical properties arising from high surface area and quantum confinement effects in two dimensions [1–3]. Therefore, the 2D nanomaterials exhibit wide potential applications in various applications, such as optoelectronic devices, energy conversion, and bio-sensing [4–9]. To date, different types of 2D compounds have been developed, and layered transition metal dichalcogenides (TMDCs) have become one of the most popular objectives due to their indirect-to-direct bandgap transition when exfoliated to monolayer and possessing intriguing optical properties [4, 9]. Therefore, it is necessary to exploit synthetic strategies to prepare TMDC nanocrystals. Till now, different synthetic approaches including chemical vapor deposition [10, 11], ion-intercalation and exfoliation [12, 13], and colloidal chemical synthesis [14, 15] have been developed for synthesis of TMDCs. Among these different synthetic methods, colloidal chemical synthesis is widely used in the preparation of ultrathin 2D nanomaterials. Although some progress has been made in the colloidal synthesis of 2D nanomaterials, some challenges still remain in this field. Therefore, it is interesting to develop a simple heating-up (non-injection) method to prepare molybdenum disulfide (MoS_2_) nanosheets, which is reliable and does not need any injection and pre-synthesis of precursors.

In this paper, a simple heating-up colloidal chemical approach has been developed to prepare MoS_2_ nanosheets, which involves the direct heating of the mixture of molybdenum precursors and 1-dodecanethiol (DDT) in the non-coordinating solvent. The crystal structure, morphology, and chemical composition of the as-obtained products were characterized by X-ray diffraction (XRD) patterns, Raman spectra, transmission electron microscopy (TEM), atomic force microscopy (AFM), and X-ray photoelectron spectroscopic (XPS) techniques. The MoS_2_ nanosheets could disperse well in the semiconducting polymer, which was used to fabricate sandwiched structured electrically bistable devices. An obvious electrical bistability was observed in the current-voltage (*I-V*) results, and the charge transport mechanism was discussed based on the organic electronic models.

## Methods

A typical synthesis MoS_2_ nanosheets is described as follows. Stoichiometric of Mo(acac)_2_, DDT, and 1-octadecene (ODE) were mixed in a 50-ml three-necked flask, and then the reaction solution was purged through nitrogen for 20 min under magnetic stirring. Afterwards, the mixture was heated to 280 °C slowly under the protection of nitrogen and kept at the temperature for 4 h. After the completion of the reaction, the reaction mixture was cooled down to room temperature naturally after removal of the heating source. The MoS_2_ nanosheets were obtained by addition of excess ethanol and centrifuged at 6000 rpm for 10 min, and then the precipitates were redispersed in chloroform. The precipitation and washing process was repeated twice, and the as-obtained products were dispersed in chloroform or dried in vacuum for next characterization.

The as-obtained MoS_2_ nanosheets were mixed with poly (N-vinylcarbazole) (PVK) in chlorobenzene, in which the weight ratio of MoS_2_ to PVK was 1:1 and the total concentration was 20 mg/mL. The electrically bistable device was fabricated as follows: the glass substrate coated with an indium-tin-oxide (ITO) anode was pre-cleaned and then the poly (3, 4-ethylenedioxy- thiophene):poly-(styrene-sulfonate) (PEDOT:PSS) was spin-coated onto the substrate as a buffer layer and then annealed at 150 °C for 15 min. Afterwards, the MoS_2_:PVK thin film was formed by using a spin-coating technique. Finally, the Al top electrodes were thermally evaporated through a shadow mask at a pressure of approximately 10^−6^ Torr.

The XRD patterns were measured by a D8 ADVANCE X-ray diffractometer. The thermogravimetric analysis (TGA) and differential thermal gravity (DTG) were taken on a PYRISI thermal gravimetric analyzer. The Raman spectrum was obtained using HR Evolution Raman spectrometer. TEM images were collected by Tecnai G2 F20 transmission electron microscope. The AFM patterns of MoS_2_ nanosheets were measured in Bruker Multimode 8 Scanning probe microscopes (SPMs). The XPS measurements were performed on an ESCALAB 250 spectrometer with a 300-W Al Ka radiation source. The *I-V* characteristics of the devices were measured by using a Keithley Source Meter 2612 controlled by a computer. All the measurements were carried out at room temperature.

## Results and Discussion

**Fig. 1:**
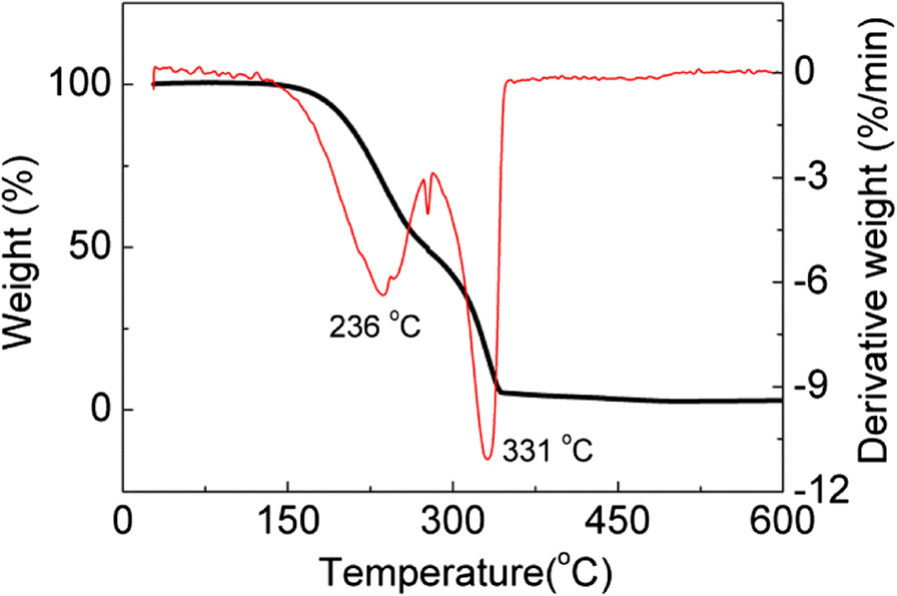
TGA and DTG curves of the Mo-thiolate precursor

To gain the decomposition information of the Mo-thiolate precursors, Fig. 1 depicts the TGA and DTG results of the precursors. As shown in the TGA curve, a rapid weight loss is observed in the elevating temperature from 150 to 350 °C, in which two steps appear at about 230 and 320 °C, respectively. Accordingly, the DTG curve displays two obvious peaks at 236 and 331 °C. Based on the previous report [16], the weight loss between 150 to 280 °C arises from the volatilization or decomposing of DDT and the other decomposition region from 280 to 350 °C may be attributed to the decomposing of ODE.

**Fig. 2:**
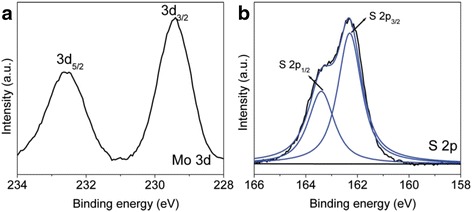
High-resolution XPS results of **a** Mo 3d and **b** S 2p of MoS_2_ nanosheets

The chemical composition and valence state of MoS_2_ nanosheets were studied by XPS spectra. Figure 2 depicts the high-resolution XPS results of Mo 3d and S 2p. As shown in Fig. 2a, two peaks located at 232.6 and 229.4 eV are observed, which can be ascribed to Mo 3d_3/2_ and Mo 3d_5/2_ of 2H-phase MoS_2_, respectively. The binding energy positions of the two peaks confirm the valence of Mo is +4, which is consistent with the previous report [17]. As can be seen in Fig. 2b, the S 2p peak can be fitted by using a spin-orbit separation of 1.1 eV, and the doublet peaks are located at 162.3 (S 2p_3/2_) and 163.4 eV (S 2p_1/2_), which are the characteristic of sulfide of the Mo–S bond [18]. The atomic ratio of S to Mo is estimated to be 2.6:1 based on the XPS results, which is higher than the stoichiometric ratio of MoS_2_, which may arise from the capping ligand of DDT.

**Fig. 3:**
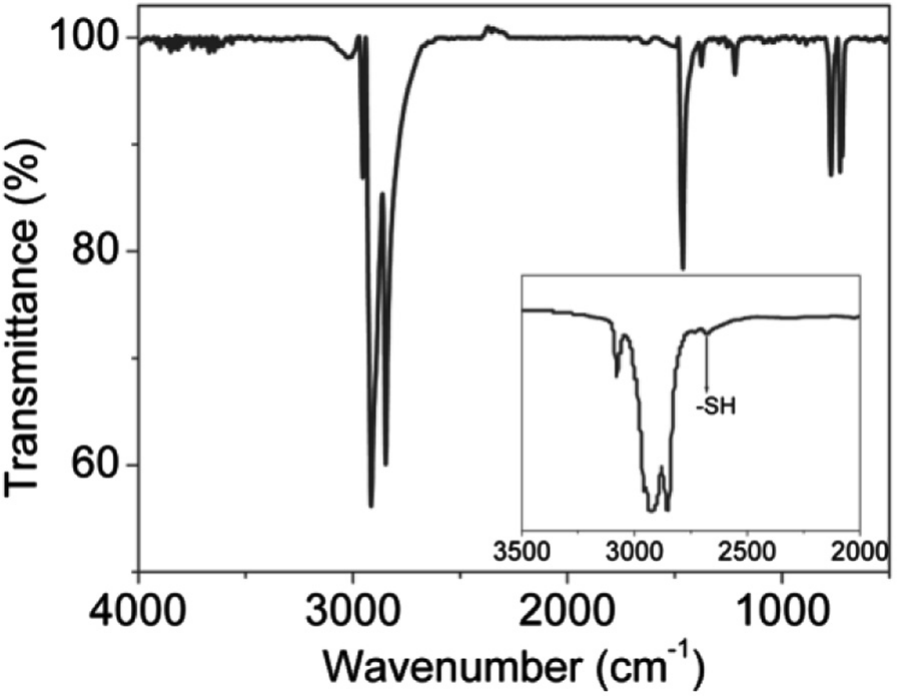
FTIR spectrum of DDT-capped MoS_2_ nanosheets. The *inset* shows the FTIR spectrum of free DDT

To further confirm the DDT coating on the surface of MoS_2_ nanosheets, the Fourier transform infrared spectroscopy (FTIR) of MoS_2_ nanosheets is given in Fig. 3. Two obvious sharp peaks at 2914 and 2846 cm^−1^ correspond to the asymmetric methyl stretching (CH_3_) and asymmetric methylene (CH_2_) stretching modes, respectively [19]. The FTIR peak at 1462 cm^−1^ belongs to the scissoring of –CH_2_–, and the two peaks at 773 and 730 cm^−1^ are the characteristic peaks of CH_3_ rocking origin [20]. As compared to the free DDT, the band at 2577 cm^−1^ disappears in the MoS_2_ nanosheets, which indicates the formation of Mo–S bond [21]. All the FTIR information confirms the DDT capping on the surface of the nanosheets.

**Fig. 4:**
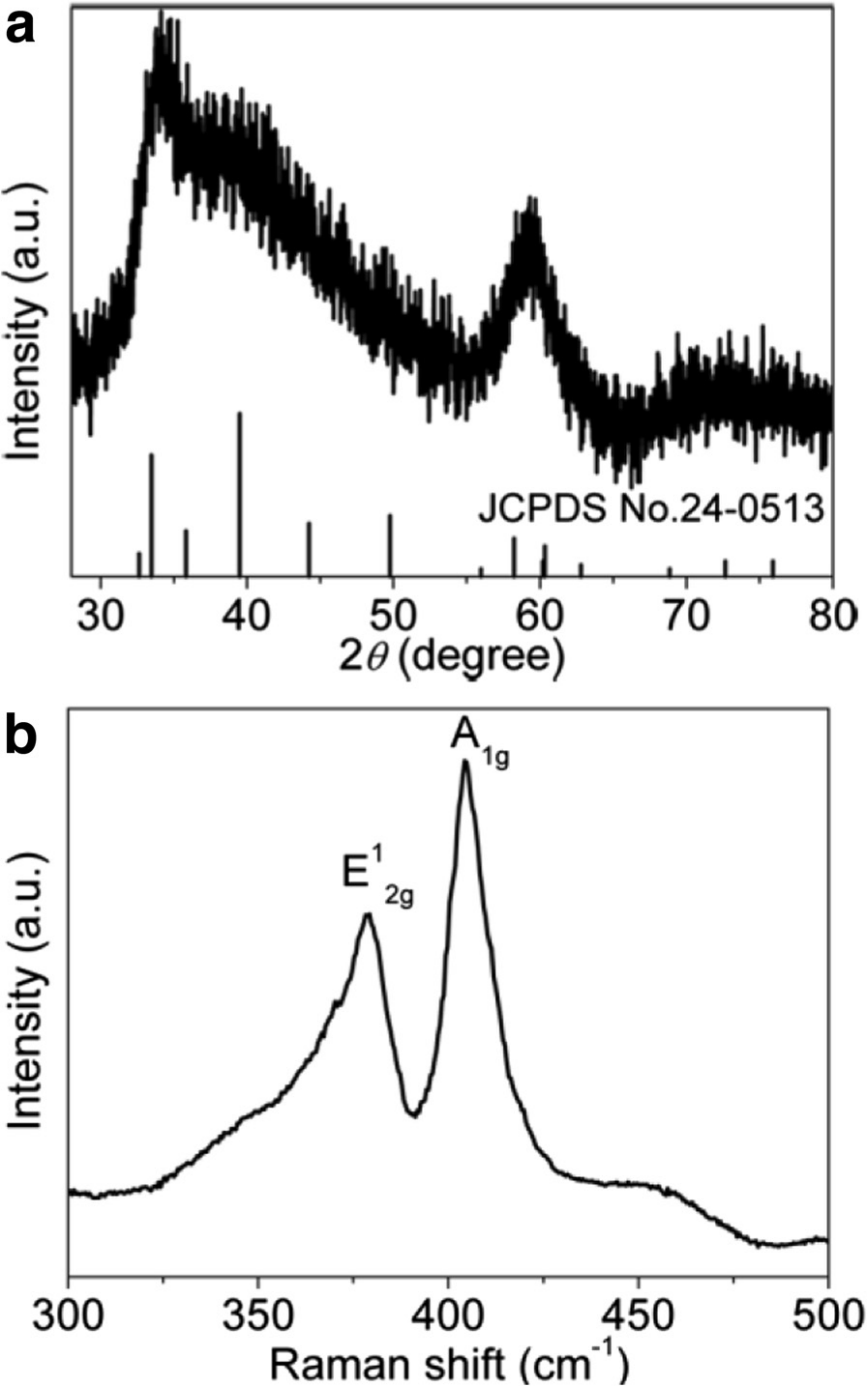
**a** XRD patterns and **b** Raman spectrum of MoS_2_ nanosheets

The crystal structure of the as-obtained MoS_2_ nanosheets has been confirmed by the XRD patterns shown in Fig. 4a, the diffraction peaks are broadened due to the nature of their very small size. All the diffraction peaks can match well with the bulk hexagonal MoS_2_ (JCPDS No. 24-0513). The Raman spectrum was used to further analyze the molecular structure, which is shown in Fig. 4b. Two strong bands are located at 379.3 and 404.8 cm^−1^, which are ascribed to the in-plane vibrational (E^1^_2g_) and the out-of-plane vibrational (A_1g_) modes, respectively [22, 23]. This is in good agreement with the previous report, which demonstrates the formation of MoS_2_.

**Fig. 5:**
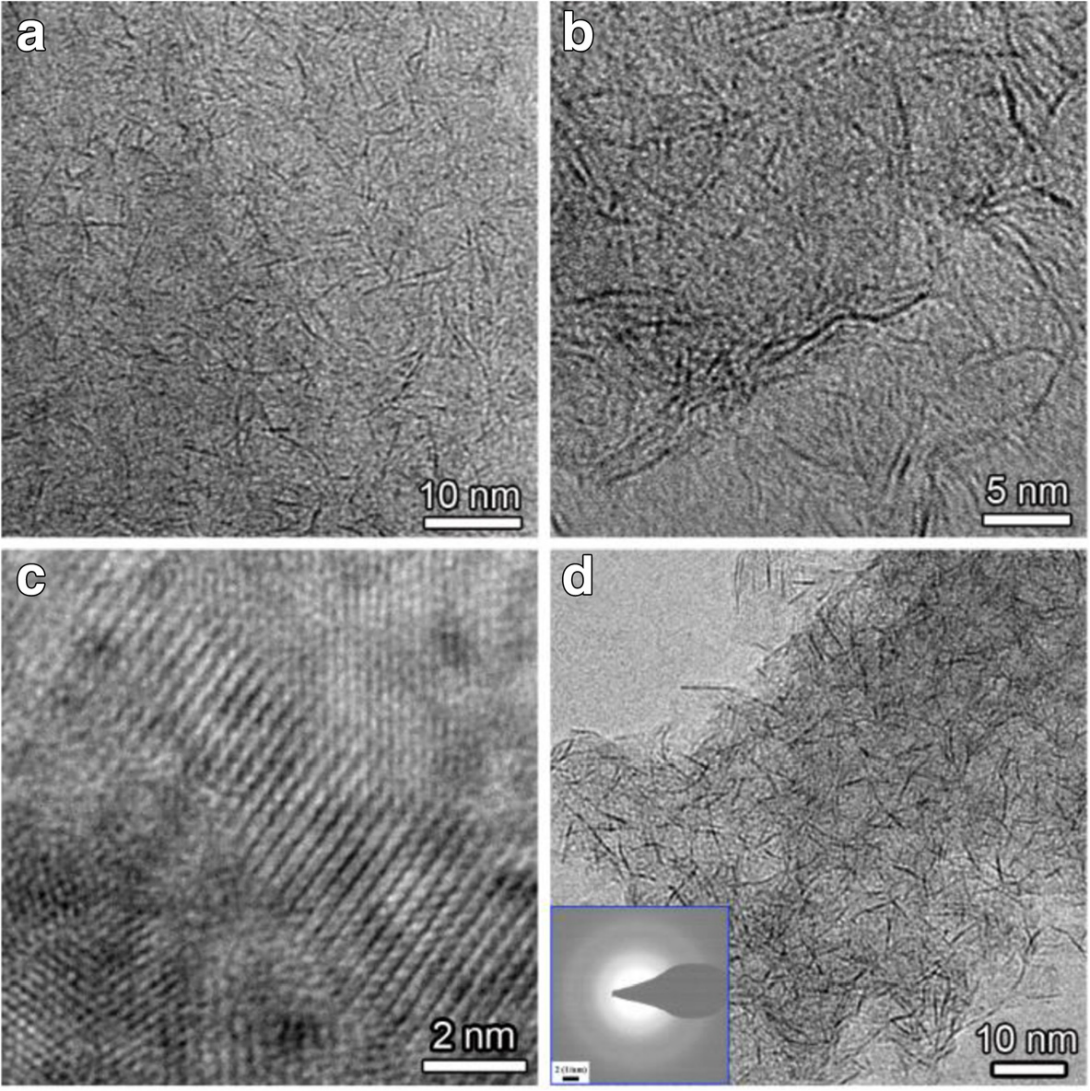
TEM images of MoS_2_ nanosheets obtained at different reaction times: **a** 30 min, **b** 120 min; **d** 240 min, and **c** the HRTEM image of MoS_2_ nanosheets obtained at 120 min

**Fig. 6:**
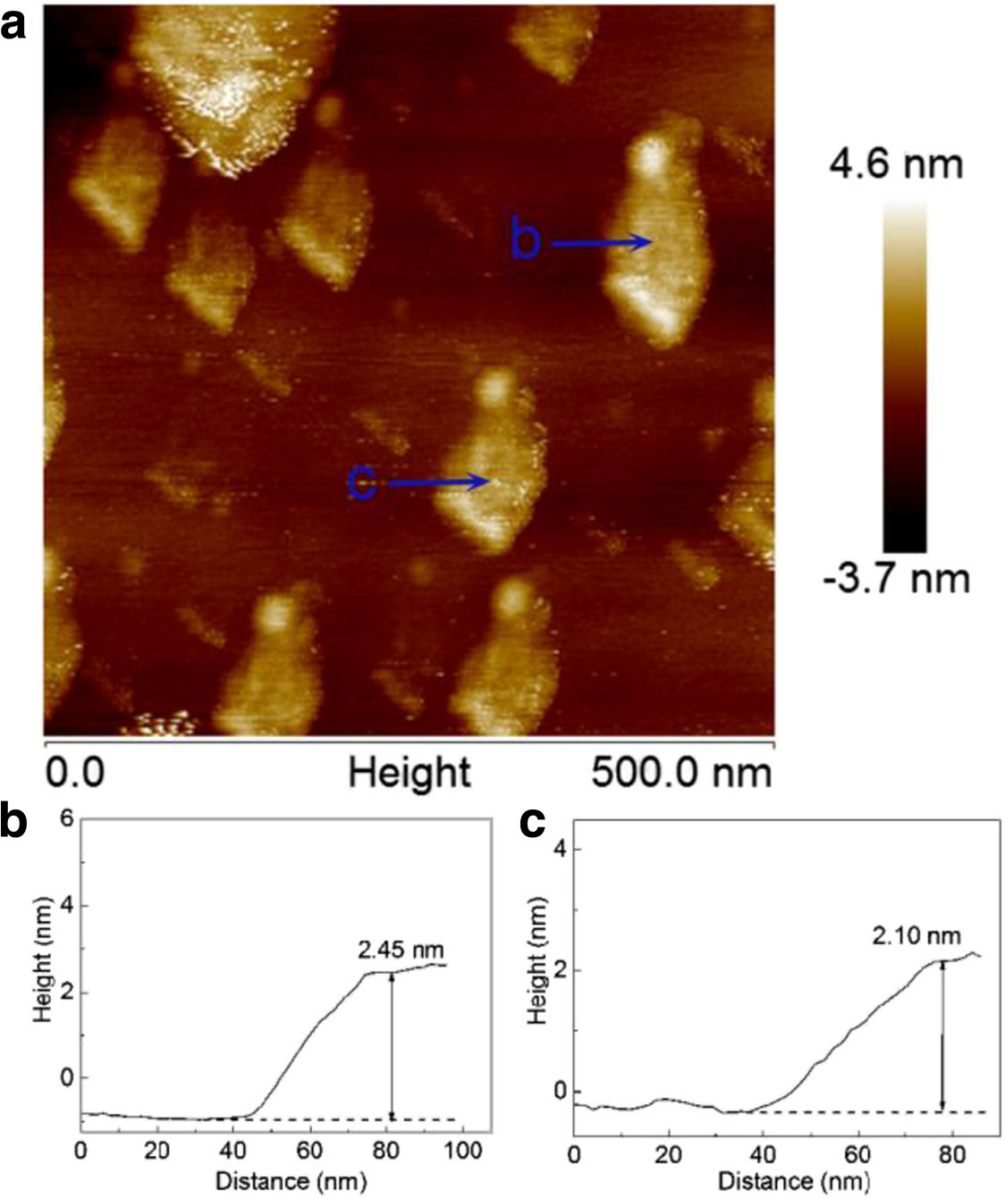
**a** A typical AFM image of MoS_2_ nanosheets obtained at 240 min and **b**, **c** the topographic height profiles of the two parts labeled in **a**

The morphology of MoS_2_ nanosheets obtained at different reaction times were characterized by the TEM images, and the TEM images are shown in Fig. 5. As shown in Fig. 5a, b, d, the as-obtained products are like nanowires at first glance; however, all the products exhibit a sheet-like morphology after careful observation, which can be confirmed by the edge of the products shown in Fig. 5d. As a matter of fact, the flexural fold is present in the product due to their ultrathin thickness of the nanosheets. The typical HRTEM image of the product obtained at 120 min shown in Fig. 5c indicates their good single crystallinity. To further confirm the morphology of nanosheets, the AFM images of the products obtained at 240 min were measured, and a typical AFM image as well as the as topographic height profile are presented in Fig. 6. The AFM images depicted in Fig. 6a indicate that the size of the nanosheet is up to hundreds of nanometers, and the height of the selected two parts is measured to be 2–3 nm, which is equivalent to 4–5 monolayer thickness for S–Mo–S structures [24].

**Fig. 7:**
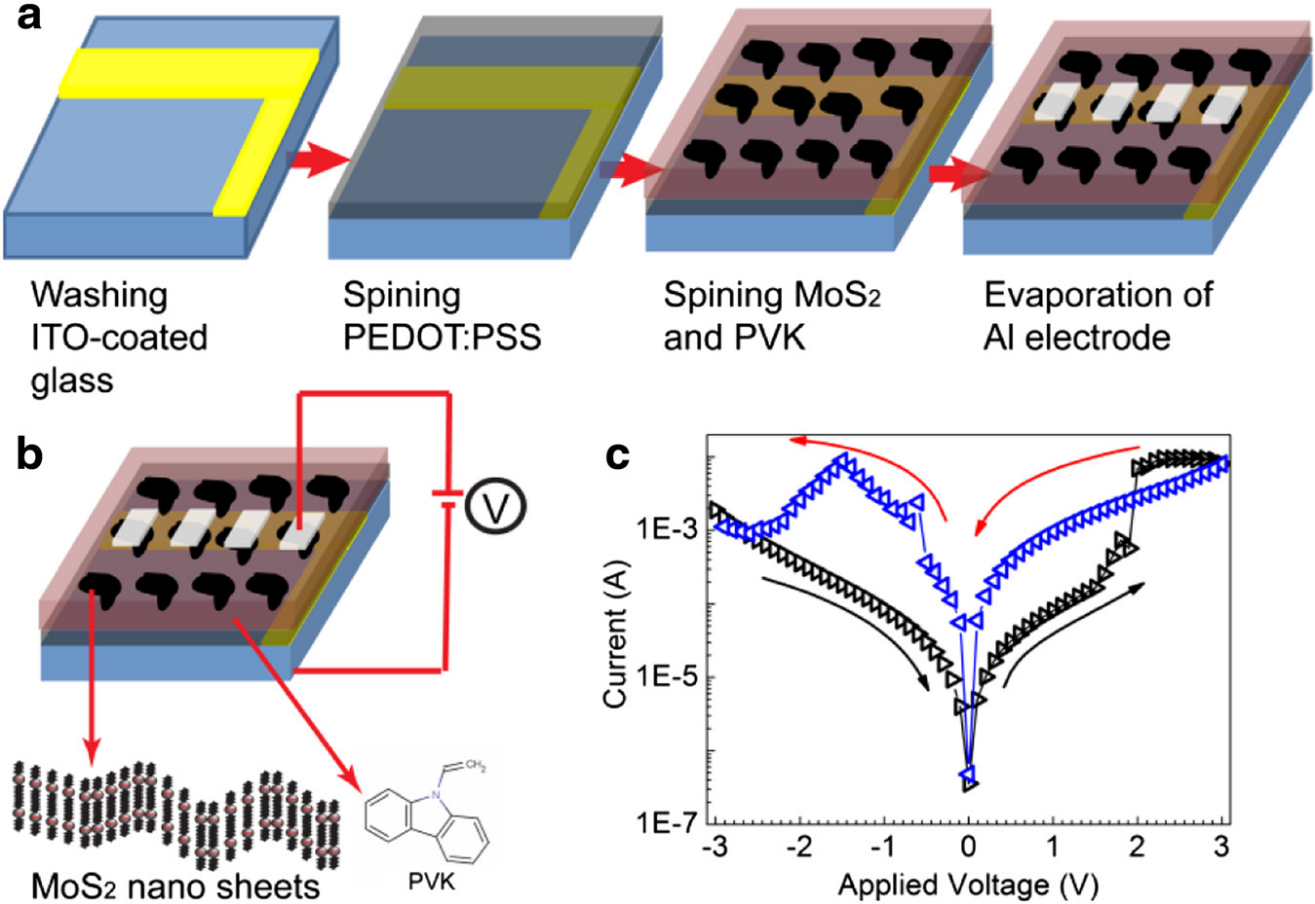
**a** Schematic illustration of the fabrication process of the electrically bistable devices, **b** device structure and the molecular structure of PVK, and **c** current-voltage of the device

Electrical bistability of the metal sulfide nanocrytals was reported in previous literatures, which often displayed two different conducting states at the same sweeping voltage [25–27]. However, the electrical bistability of the MoS_2_ nanosheets was rarely reported. Herein, a sandwiched electrically bistable device was fabricated based on the blending film composed of MoS_2_ nanosheets and PVK by using a simple spin-coating method, in which the MoS_2_ nanosheets was dispersed in PVK matrix to make the film more smooth. The schematic illustration of the device fabrication process and the device structure is given in Fig. 7a, b. As shown in Fig. 7c, an obvious electrical hysteresis is observed when the sweeping voltage is scanned from −3 to 3 V and then from 3 to −3 V, which exhibits a high-conducting state (ON state) and a low-conducting state (OFF state) at the same sweeping voltage. Such an electrical hysteresis behavior is an essential feature for the electrically bistable device. As the sweeping voltage is scanned from 0 to 3 V, the current is increased rapidly when the sweeping voltage exceeds about 2 V, which indicates that the conducting state transforms from an OFF to ON state. When the sweeping voltage scans from 0 to −3 V, the current reaches its maximum at about −1.5 V, and then decreases quickly with the reverse voltage increasing, which is a typical negative differential resistance (NDR) behavior. As a result, the conducting state changes from an ON to OFF state. As stated in our previous report [27], the electrical bistability is not observed in the device based on only the PVK film, which indicates that the MoS_2_ nanosheets play a significant role in the electrical bistability.

**Fig. 8:**
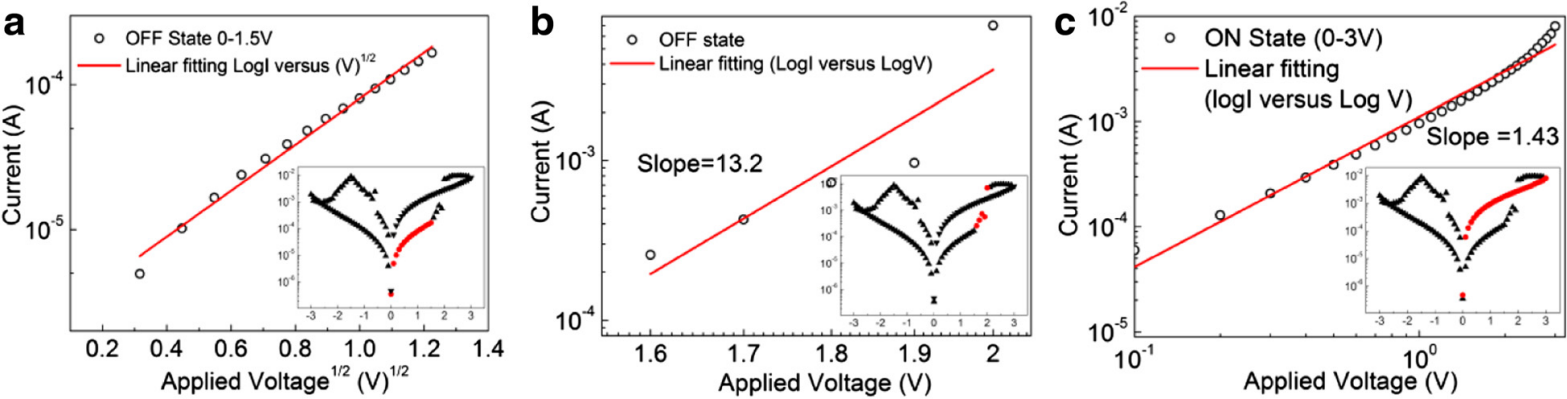
Experimental data (*open cycle*) and theoretical fitting curve (*solid line*) of *I-V* characteristics in the positive voltage region of electrically bistable devices: **a** relationship between log*I* and log*(V)* ^*1/2*^ in the region of 0–1.5 V (OFF state); **b** relationship between log*I* and log*V* in the region of 1.5–2 V (OFF state); and **c** relationship between log*I* and log*V* in the region of 0–3 V (ON state)

In order to understand the switching and the charge transport mechanism of the electrically bistable device based on MoS_2_ nanosheets and PVK, Fig. 8 shows the experimental and fitting data of the *I-V* curves in the region of positive voltage. Generally, the carrier transport mechanism of the electrically bistable device can be described by space charge-limited current (SCLC) and trap-controlled space charge-limited current (TCLC) [25], thermionic emission (TE) [26], and ohmic conduction model [27]. As shown in Fig. 8a, the log*I* is in proportion to *V*^*1/2*^ in the region from 0 to 1.5 V of the OFF state, which matches well with the TE model. However, when the voltage increases from 1.5 to 2 V for the OFF state, the relationship between log*I* and log*V* can be fitted by a line with a slope of 13.2, which is attributed to the TCLC conduction model in this region (Fig. 8b). When the conduction state transits from an OFF to ON state, the experimental data (log*I* versus log*V*) in the sweeping voltage region of 0−3 V can be fitted by a straight line with a slope of 1.43, which is close to the ohmic model (Fig. 8c).

## Conclusions

In summary, a simple heating-up colloidal approach was developed to prepare MoS_2_ nanosheets, which was synthesized in DDT and non-coordinating solvent. The as-obtained products had a sheet-like morphology, whose crystal structure was characterized by the XRD patterns and Raman spectrum. An electrically bistable device was fabricated based on the blends of MoS_2_ nanosheets and PVK, and an obvious electrical bistability and NDR behavior was observed in the *I-V* curves. The charge transport could be described in terms of the organic electronic models, and the charge transport mechanism changed from the thermionic emission to the ohmic model during the transition of the conducting state from an OFF state to ON state. The result indicates that the MoS_2_ nanosheets may have potential application in the organic/inorganic hybrid electrically bistable devices.
